# Anomalous Release Kinetics of Prodigiosin from Poly-N-Isopropyl-Acrylamid based Hydrogels for The Treatment of Triple Negative Breast Cancer

**DOI:** 10.1038/s41598-019-39578-4

**Published:** 2019-03-07

**Authors:** Y. Danyuo, C. J. Ani, A. A. Salifu, J. D. Obayemi, S. Dozie-Nwachukwu, V. O. Obanawu, U. M. Akpan, O. S. Odusanya, M. Abade-Abugre, F. McBagonluri, W. O. Soboyejo

**Affiliations:** 1 Department of Mechanical Engineering, Ashesi University, Berekuso, Ghana; 2 Department of Materials Science and Engineering, African University of Science and Technology, km 10, Airport RD, Federal Capital Territory, Abuja, Nigeria; 3 Department of Theoretical and Applied Physics, African University of Science and Technology (AUST), Km 10, Airport Road, Federal Capital Territory, Abuja, Nigeria; 4 Department of Mechanical Engineering, Worcester Polytechnic Institute, Higgins Labs, 100 Institute Road, Worcester, MA 01609 USA; 5 Biotechnology Advance Research Center, Sheda Science and Technology Complex (SHESTCO), Abuja, Federal Capital Territory Nigeria; 6 Department of Materials Science and Engineering, Kwara State University, Malete, Nigeria; 7 Department of Mechanical Engineering, Academic City College, Accra, Ghana

## Abstract

This paper presents the anomalous release kinetics of a cancer drug (prodigiosin) frompoly-n-isopropyl-acrylamide **(**P(NIPA))-based gels. The release exponents, n, which correspond to the drug release mechanisms, were found to be between 0.41 and 1.40. This is within a range that include Fickian case I (n = 0.45) and non-Fickian diffusion (case II) (n > 0.45) for cylindrical drug-loaded structures. The results, however, suggest that the release exponents, n, correspond mostly to anomalous case II and super case II transport mechanics with sigmoidal characteristics. The drug release kinetics of the P(NIPA)-based hydrogels are well described by bi-dose functions. The observed drug release behavour is related to the porosity of the hydrogels, which can be controlled by cross-linking and copolymerization with acrylamide, which also improves the hydrophilicity of the gels. The paper also presents the effects of cancer drug release on cell survival (%), as well as the cell metabolic activities of treated cells and non-treated cells. The implications of the results are discussed for the development of implantable thermosensitive gels for the controlled release of drugs for localized cancer treatment.

## Introduction

In recent years, significant progress has been made in the development of drug delivery devices^1–4^, for localized cancer treatment^5–8^. Such devices have also been shown to have the potential to reduce the side effects associated with bulk chemotherapy and radiotherapy^9,10^. There is, therefore, a need to explore drug delivery approaches that enable the localized delivery of cancer drugs to tumor sites^11^, since, less than ∼1% of cancer drugs reach the desired tumor sites during bulk systemic chemotherapy^12^. In contrast, implantable biomedical devices have been shown to have the potential to deliver cancer drugs to specific tumor sites^3^. Such devices, which can be inserted during tumor removal/surgery, can be designed to release cancer drugs to specific tumor sites or remaining cancer cells/tissue that surround the device^6,10,13^. However, there is a need to understand the drug release kinetics associated with implantable devices for the controlled release of cancer drugs^5,6,14^.

Thermo-sensitive hydrogels are characterized by a lower critical solution temperature (LCST), a phase transition temperature beyond which the gel collapses to allow drug elution^6^. In the case of poly-n-isopropyl-acrylamide (P(NIPA))-based hydrogels, significant efforts have been made to study cancer drug release from such gels due to the potential to use their thermo-sensitivity in the triggering of controlled release^3,15^. The release kinetics of drugs from P(NIPA) helps in the selection of a copolymer hydrogel with an appropriate LCST that can enhance drug release even at physiological conditions. However, recent work^6^ has revealed that their cancer drug release mechanisms are non-Fickian^6,7^. There is, therefore, a need to develop a better understanding of the non-Fickian drug release mechanisms of P(NIPA)-based hydrogels. This will be explored in the current study using a combination of drug release experiments, mathematical fitting of drug release data, and studies of the morphology and porosity of P(NIPA)-based hydrogels with different levels of acrylamide copolymers. The implications of the results are also discussed for the development of implantable devices for localized cancer treatment.

## Results

### Morphology and De-swelling Kinetics

**Figure 1:**
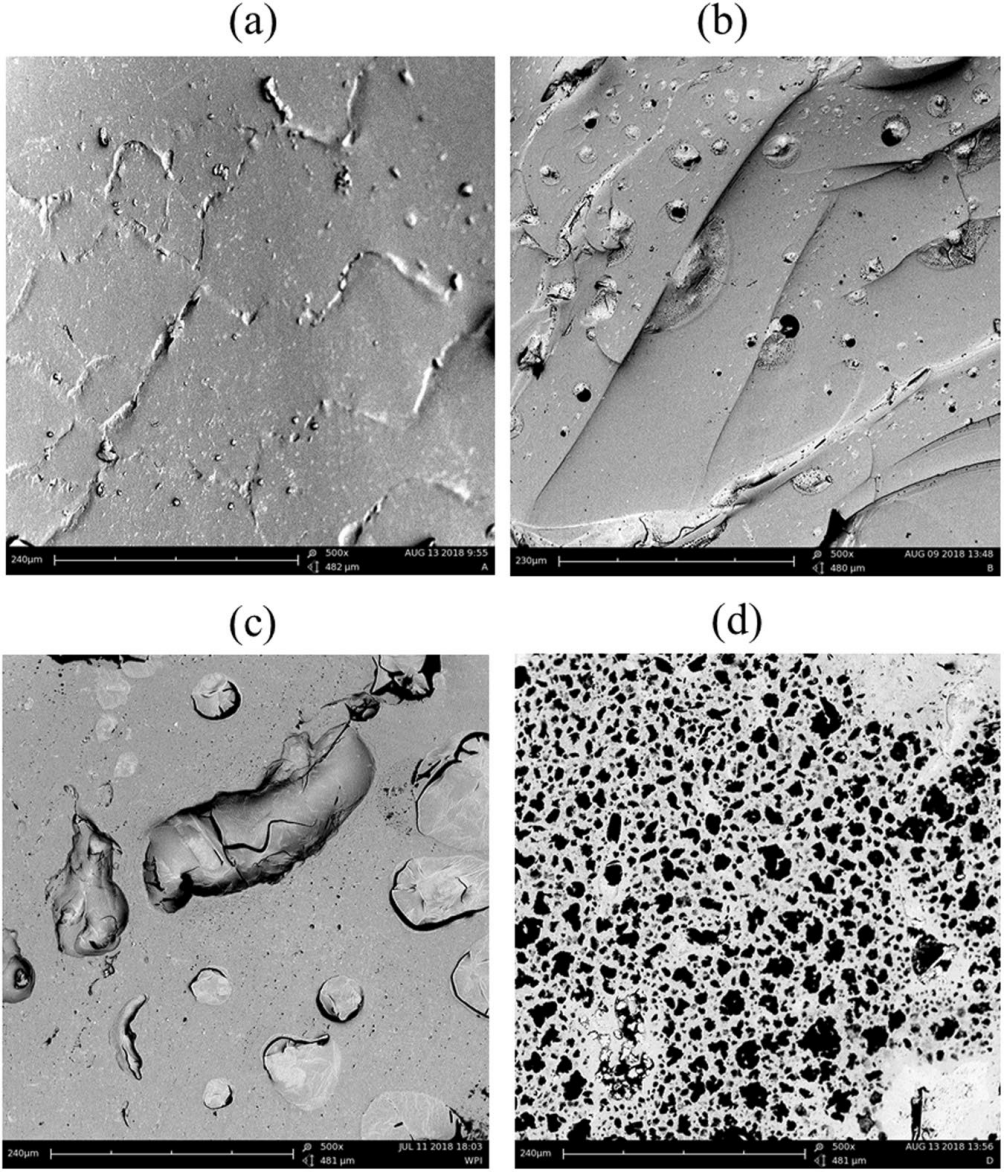
SEM Micrographs Showing the Effect of Copolymerization on Gel Morphology: (**a**) Homopolymer, (**b**) P(NIPA)-co-AM (95:5 wt.%), (**c**) P(NIPA)-co-AM (90:10 wt.%) and (**d**) P(NIPA)-co-AM (85:15 wt.%). Scale Bar ~481 *μm*.

The microstructures of the gels (Fig. 1a–d) present the effects of copolymer ratio on the morphology of P(NIPA)-based copolymers. The homopolymer matrix (Fig. 1a) indicates less porosity with revealing collapsed walls. The result in Fig. 1c clearly shows the structures of the polymers with micro-cracks and micro-pores. The pore diameters of the homopolymer ranged from 1.67–39.20 μm with an average pore diameter of 9.62 μm (Fig. 1a). This hyddrogel structure also revealed interconnecting cracks with crack lengths ranging from 17.77–84.52 μm (average crack length ∼48.70 μm). In the case of P(NIPA)-based co-polymer matrices (Fig. 1b–d), we observed a predominance of micro-pores at multiple locations with increasing amount of acrylamide. P(NIPA)-co-AM (95:5 wt.%) (Fig. 1c) exhibited pore diameters between 0.1 and 36.07 *μm* (with mean diameter of ∼7.77 *μm*, whereas P(NIPA)-co-AM (90:10 wt.%) (Fig. 1c) had collapsed cracks between 0.01–121.01 *μm* (with an average pore diameter of 61.87 *μm*). The entire structure of P(NIPA)-based co-polymer matrices (85:15 mol%) was highly porous, characterized by micro-voids and micro-pores with pore diameters between 0.05 and 82.07 μm (with an average pore diameter of 25.59 μm) (Fig. 1d).

**Figure 2:**
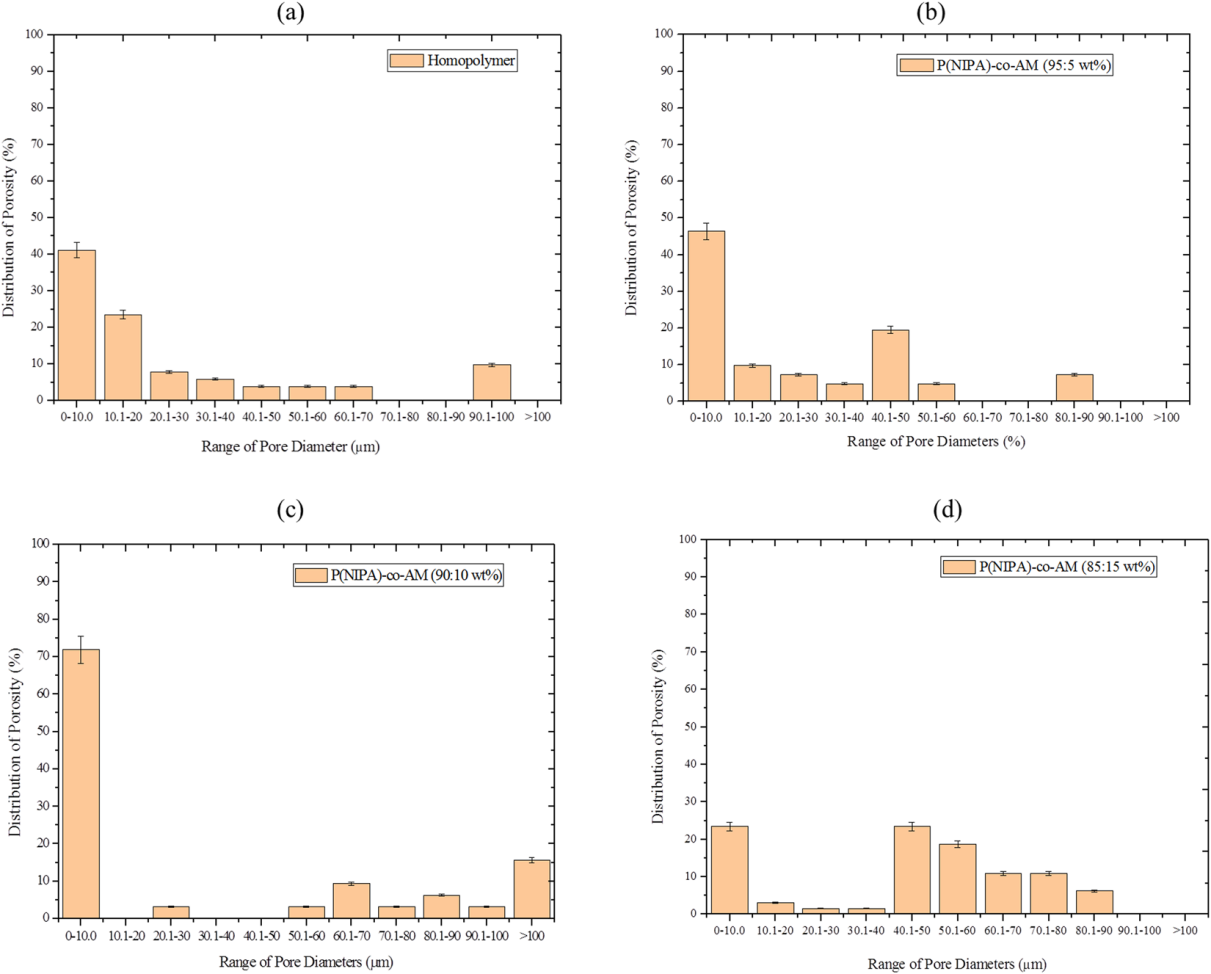
Effect of Copolymerization on the Distribution of Pore Diameters of P(NIPA)-Based Polymers: (**a**) Homopolymer, (**b**) P(NIPA)-co-AM (95:5 wt%), (**c**) P(NIPA)-co-AM (90:10 wt%) and (**d**) P(NIPA)-co-AM (85:15 wt%).

Furthermore, distribution of pore diameters from the SEM micrographs are presented (Fig. 2). The result shows the dominance and range of pore sizes within the micrographs for the different experimental samples considered. Although large pore diameters promote diffusion, the pore volume and area equally play key roles in the diffusion process of cancer drugs. The result, therefore, complements the SEM micrographs (Fig. 1). In all cases, 0–10.0 *μm* pore diameter range dominated within the micrographs presented. To some extent, the distribution of pore diameters for P(NIPA)-based hydrogels presented (Fig. 2) were skewed to the right (homopolymer and P(NIPA)-co-AM (95:5 wt%). Thus, micro pores were predominant as compared to large pore diameters. The pore distribution of P(NIPA)-co-AM (85:15 wt%) suggest a normally distributed pores with predominant pore ranges from 0–10, 40.1–50, 50.1–60, 60.1–70, 70.1–80 *μm* yielding percentage pore distributions of 23.44, 23.43, 18.75, 10.93 and 10.94, respectively. Although porosity was expected to increase with the amount of AM in the hydrogels, the porosity of P(NIPA) (90:10 wt%) was less than that of the hompolymer but the pores had larger diameters. The addition of AM generally shifted the pores sizes distribution from smaller diameters to larger diameters and this in turn enhanced drug diffusion in the copolymer hydrogels with increasing amount of AM. Hence, AM makes the sample more hydrophilic and susceptible to water/drug solutions.

The characteristics (porosity, collapsingwalls, and micro/macro cracks) of porous structures presented enable the gels to easily collapse and expand, especially when environmental temperatures are near or exceed the lower critical solution temperature with the application of an external stimulus such as heat. Thus, the heat trigger mechanism can be used to regulate drug release from P(NIPA)-based hydrogels.

**Figure 3:**
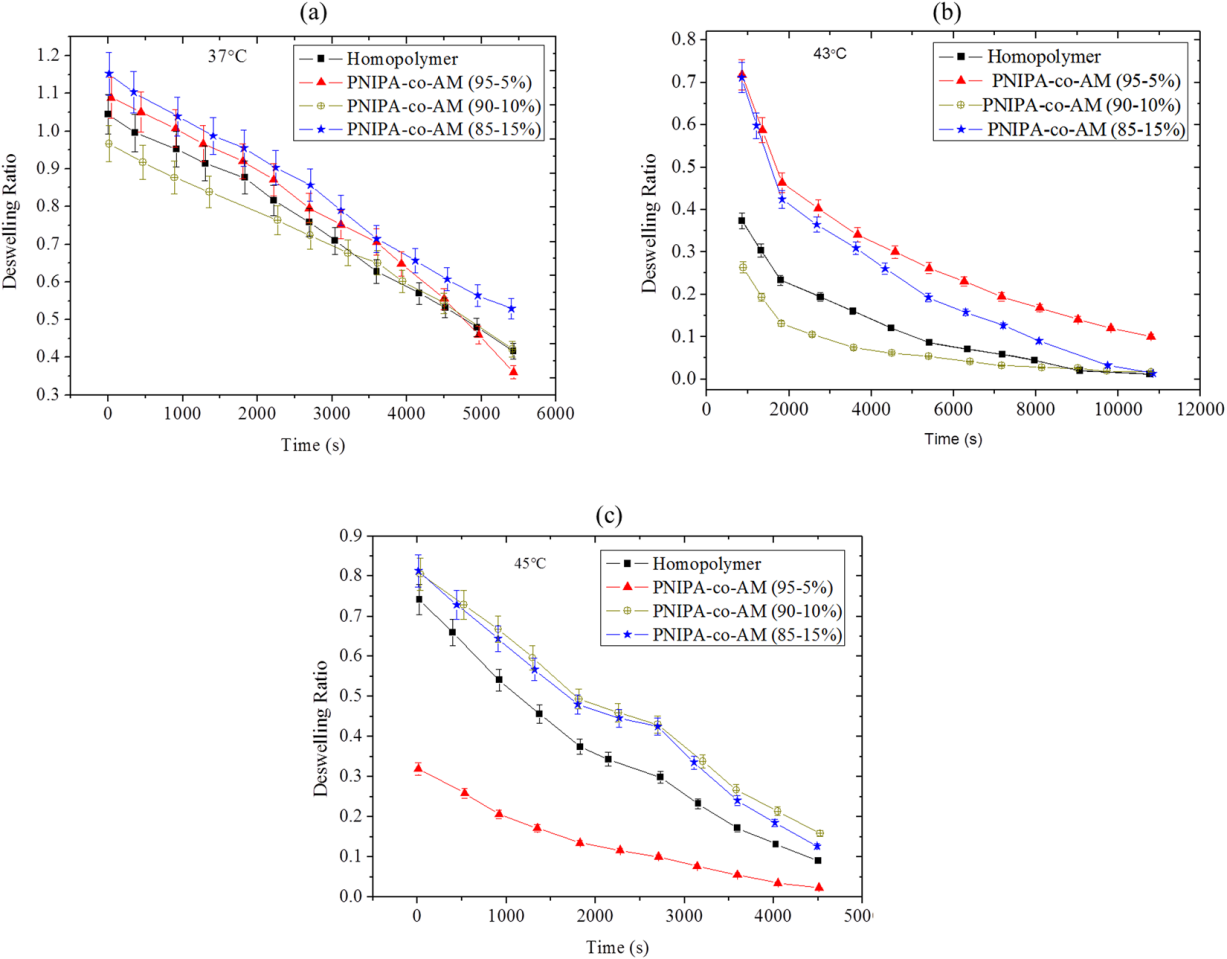
De-swelling Ratios of Prodigiosin Released from P(NIPA)-Based Hydrogels at: (**a**) 37 °C, (**b**) 43 °C and (**c**) 45 °C.

The swelling index and water absorbability of P(NIPA)-based hydrogels are presented in Table 1. Co-polymerization of P(NIPA) with acrylamide increased the absorption ability of the PG drug in the hydrogel from 1400–2820% (within 72 h). Furthermore, the plots of the de-swelling ratio against time revealed differences in the de-swelling characteristics at 37, 43 and 45 °C (Fig. 3a–c).

**Table 1:** Absorbability and Swelling Index (H-index).

Gel Code	Composition of Gel (NIPA: AM (w%/wt%))	Water Absorbability (%)	Equilibrium in Deionized water	Equilibrium 70% Ethanol	H-index (100%)
A	100:00	1400.00	14.90	7.00	47.00
B	90:50	2400.00	24.00	12.00	50.00
C	90:10	2660.00	26.60	14.00	52.60
D	85:15	2820.00	28.20	15.80	56.74

Gel codes: ^A^Homopolymer, ^B^P(NIPA)-co-AM (95:5 wt.%), ^C^P(NIPA)-co-AM (90:10 wt.%) and^D^ P(NIPA)-co-AM (85:15 wt.%).

### Order of Drug Release

**Figure 4:**
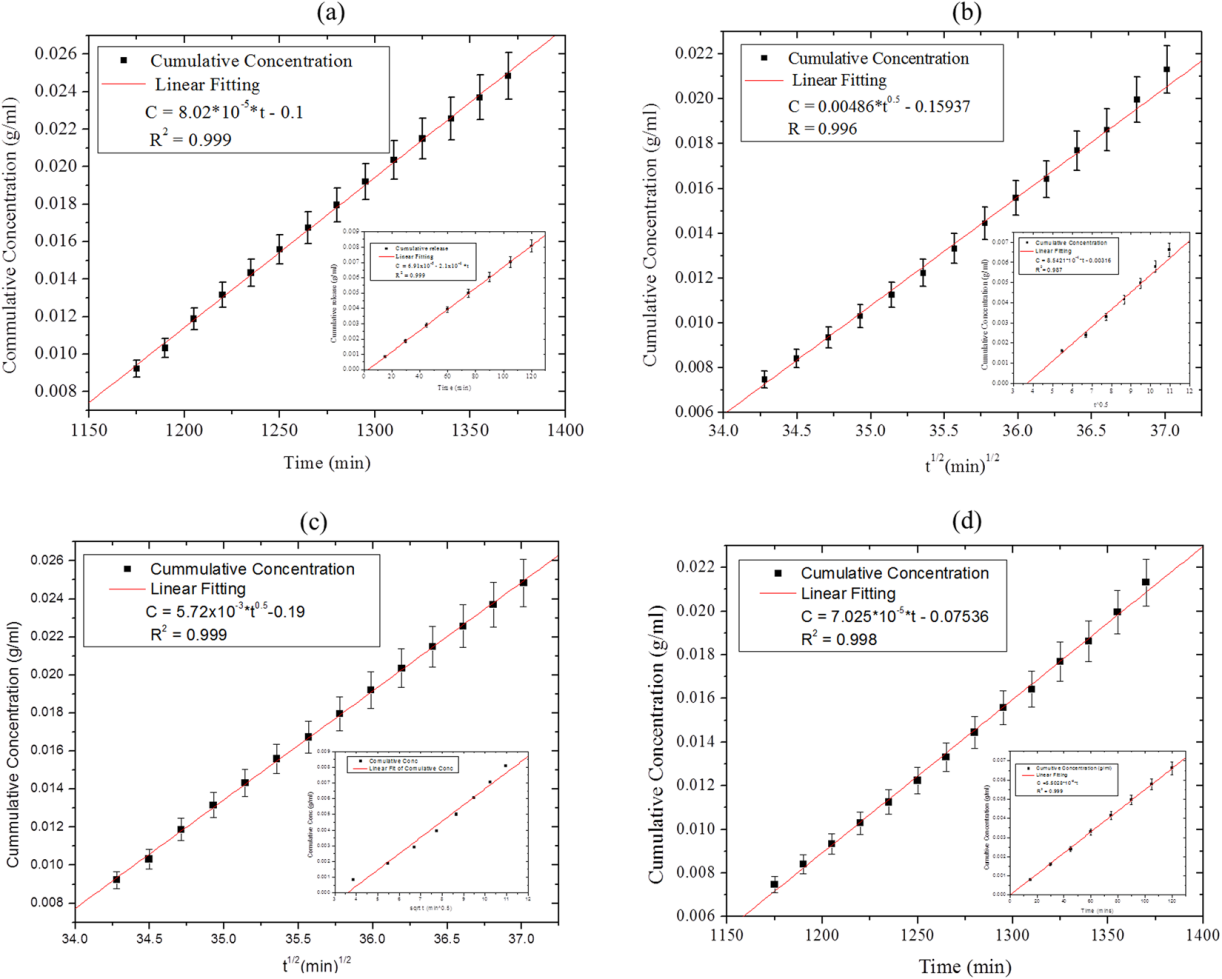
Estimation of Drug Release Models for P(NIPA)-homopolymer: (**a**) and (**c**) Satisfied Zeroth Order and Huguchi Models, while (**b**) and (**d**) Satisfied Huguchi Models for P(NIPA-co-AM) (95:5 wt%).

The order of drug release kinetics was determined by plotting the cumulative concentration of drug against $t\,{\rm{or}}\,{t}^{\frac{1}{2}}$ (Fig. 4a–d). In the case of the P(NIPA) homopolymer, linear plots in Fig. 4a and c showed that the homopolymer exhibited zeroth order kinetics with *R*^2^ = 0.999. However, linear plots of cumulative concentration versus ${t}^{\frac{1}{2}}$ (Fig. 4b and c) show that the homopolymer satisfied the Higuchi model (*R*^2^ = 0.999). Hence, the drug release occurs at a constant rate that is independent of drug concentration (zeroth-order model). However, the Higuchi model^16^ assumes that the swelling and dissolution of drugs in the polymer matrix are negligible, while the drug diffusivity is constant. This implies that drug diffusion occurs in one dimension^17^.

### Mechanisms and Order of Drug Release

**Figure 5:**
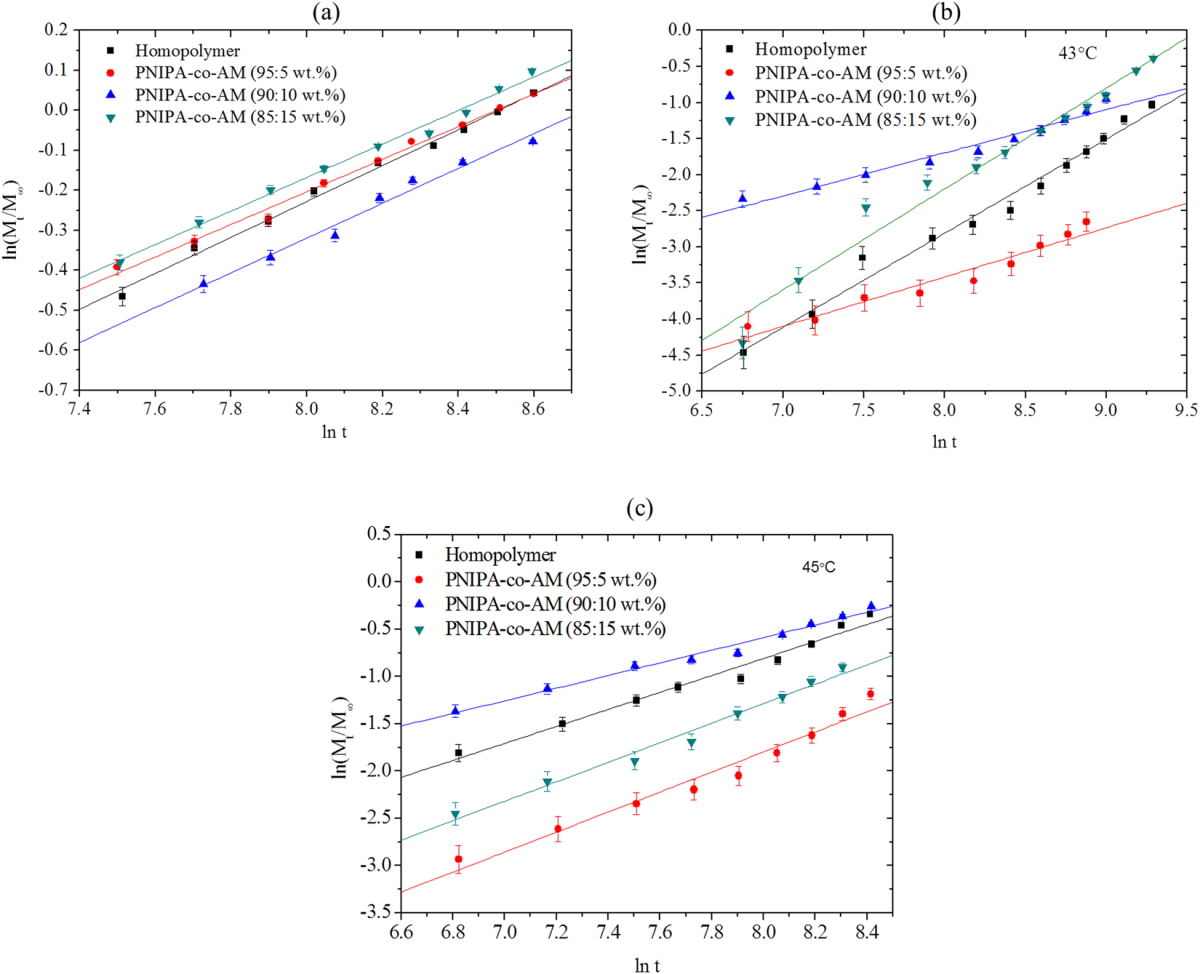
Determination of Drug Release Exponent, n, for P(NIPA)-Based Hydrogels at: (**a**) 37 °C, (**b**) 43 °C and (**c**) 45 °C.

The drug release exponents, n, and the diffusion coefficients, D, were obtained from plots of ln(*M*_*t*_/*M*_∞_) versus In *t*, shown in Fig. 5a–c for P(NIPA)-based hydrogels that were loaded with PG. The results of drug release exponents, n, diffusion coefficients are presented in Tables 2 and 3, respectively. Note that, for a cylindrical geometry, an n value of 0.45 corresponds to a Fickian diffusion mechanism, i.e. diffusion-controlled transport. Anomalous transport (non-Fickian transport mechanism) is characterized by n-values that are greater than 0.45 but less than 0.89 (i.e. 0.45 < n < 0.89). Also, an n value of 0.89 corresponds to a case-II transport mechanism. A case-II anomalous diffusion process isassociated with controlled swelling. Moreover, n values greater than 0.89 are associated with a super-case-II transport mechanism.

**Table 2:** Summary of Drug Release Exponents, n, at Different Polymer Ratios and Temperatures.

Polymer Ratio	Drug Release Exponents, n and Regression Coefficient, R^2^					
	37 °C		43 °C		45 °C	
	n	R^2^	n	R^2^	n	R^2^
Homopolymer	0.45*	0.995	1.30^***^	0.982	0.90^***^	0.975
PNIPA-co-AM (95:5 wt.%)	0.41	0.993	0.68^**^	0.940	1.06^***^	0.962
PNIPA-co-AM (90:10 wt.%)	0.45*	0.978	0.56^**^	0.976	0.67^**^	0.983
PNIPA-co-AM (85:15 wt.%)	0.42	0.993	1.40^***^	0.971	1.03^***^	0.982

*Controlled Diffusion, ^**^Anomalous Transport, ^***^Super-Case II Transport Mechanism, R^2^ = Regression Coefficient (the goodness of fit to the model).

**Table 3:** Diffusion Coefficient, D and Geometric Factor, k, at Different Polymer Ratios and Temperatures.

Polymer Ratio	37 °C		43 °C		45 °C	
	k	D (m/s^2^)	k	D(m/s^2^)	k	D(m/s^2^)
Homopolymer	0.011	6.772 × 10^−8^	0.125	7.808 × 10^−7^	0.607	3.791 × 10^−6^
PNIPA-co-AM (95:5 wt.%)	0.012	7.544 × 10^−8^	0.038	2.399 × 10^−7^	0.654	4.086 × 10^−6^
PNIPA-co-AM (90:10 wt.%)	0.081	5.044 × 10^−7^	0.221	1.381 × 10^−6^	0.563	3.517 × 10^−6^
PNIPA-co-AM (85:15 wt.%)	0.014	8.826 × 10^−8^	0.064	3.995 × 10^−7^	0.662	4.138 × 10^−6^

^k^Geometric constant, ^D^Diffusion coefficient.

The results presented in Table 2, suggest that Fickian diffusion occured in the P(NIPA)-based homopolymer and P(NIPA)-co-AM (90:10 wt.%) at 37 °C with n values of 0.45. The release exponents, n, obtained for P(NIPA)-co-AM (95:5 wt.% at 43 °C)was 0.68, whiles P(NIPA)-co-AM (90:10 wt.%) at 43 and 45 °C had n values of 0.56 and 0.67, respectively. These correspond to anomalous diffusion mechanisms.

The anomalous transport class was further investigatedand was found to be dominated by a sigmoidal function^6^ with a goodness of fit, R^2^ = 0.970. Among the classes of sigmoidal functions investigated, the bi-dose response function gave higher R^2^ values than the dose-response function^18^ and the sigmoidal Richards’s second function^19^. The bi-dose response function obtained for the sigmoidal equation is given by:

1 $$y={A}_{1}+({A}_{2}-{A}_{1})\,[\frac{P}{1+{10}^{({L}_{1}-x){h}_{1}}}+\frac{1-P}{1+{10}^{({L}_{2}-x){h}_{2}}}]\,p\ne 1,\,{A}_{2} > {A}_{1}$$

where $\,{\rm{y}}=(\frac{{M}_{t}}{{M}_{\infty }})$ (i.e. the fraction of drug release), $x\,=tim{e}^{\frac{1}{2}}$, *A*_1_ is the least amount of drug release fraction $\,(\frac{{M}_{t}}{{M}_{\infty }})$, *A*_2_ is the higher value of $(\frac{{M}_{t}}{{M}_{\infty }})$, *h*_1_ and *h*_2_ are the respective first and second gradients of the sigmoidal plots. The parameters of the bi-dose response function are summarized in Table 4 (37 °C) and Table 5 (at different temperatures, 43 and 45 °C).

**Table 4:** Data from Sigmoidal Fitted Model for a Bi-Dose Response Function for P(NIPA)-Based Hydrogels at 37 °C.

Parameter	P(NIPA-co-AM) (95:5 wt.%)	P(NIPA-co-AM) (85:15 wt.%)
	Value	Value
A_1_	0.326	0.510
A_2_	1.110	1.146
L_1_	26.905	48.633
L_2_	60.251	69.465
h_1_	0.058	0.046
h_2_	0.053	0.270
p	0.998	0.818
R^2^	0.998	0.999

R^2^ = Regression Coefficient (the goodness of fit of the model).

**Table 5:** Data from Sigmoidal Fitted Model Equation for a Bi-Dose Response Function for P(NIPA)-Based Hydrogels at 43 and 45 °C.

Parameter	43 °C	43 °C	45 °C
	PNIPA-co-AM (95:5 wt.%)	PNIPA-co-AM (90:10 wt.%)	PNIPA-co-AM (90:10 wt.%)
	Value	Value	Value
A_1_	0.016	0.043	0.146
A_2_	15.097	36.642	0.791
L_1_	181.900	252.376	35.445
L_2_	98.015	98.824	59.591
h_1_	0.025	0.013	0.057
h_2_	0.417	0.258	0.130
p	0.996	0.996	0.538
R^2^	0.999	0.999	0.997

R^2^ = Regression Coefficient (the goodness of fit of the model).

**Figure 6:**
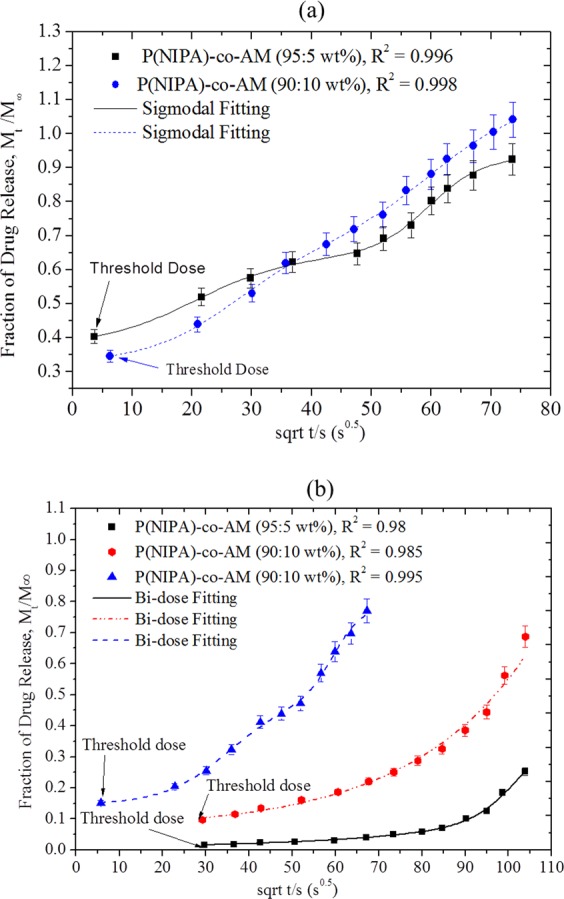
(**a**) Bi-Dose Response of a Sigmoidal Function at 37 °C, (**b**) Bi-Dose Response of a Sigmoidal Function for; P(NIPA)-co-AM (95:5 wt.%) at 43 °C, P(NIPA)-co-AM (90:10 wt.%) at 43 °C and P(NIPA)-co-AM (90:10 wt.%) at 45 °C, respectively.

It is of interest to note that the values of n obtained for P(NIPA)-co-AM (95:5 wt.%) and P(NIPA)-co-AM (85:15 wt.%) (at 37 °C) were 0.41 and 0.42, respectively. Although these n values are close to those of Fickian diffusion, they are more clearly less than 0.45. Furthermore, the anomalous n values also exhibited a sigmoidal class of diffusion. The fitted bi-dose sigmoidal plots (S-shape) are shown (Fig. 6a). Sigmoidal anomalous transport was also observed for P(NIPA)-based hydrogels copolymerized with 5 wt.% AM(P(NIPA)-co-AM (95:5 wt.%)) during release at 45 °C. These had an n value of 0.67 (Table 2).

However, super case-II transport mechanisms were reported for: the homopolymer(at 43 °C and 45 °C); P(NIPA)-co-AM (85:15 wt.%) at 43 °C; P(NIPA)-co-AM (95:5 wt.%) at 45 °C, and P(NIPA)-co-AM (85:15 wt.%) at 45 °C. These had n-values of 1.30, 1.40, 0.90, 1.06 and 1.03, respectively. These correspond to super case-II transport withn values greater than 0.89. They also suggest a rapid drug release in the hyperthermictemperatureregime of ∼43–45 °C.

### *In vitro* Effect of Drug Release

**Figure 7:**
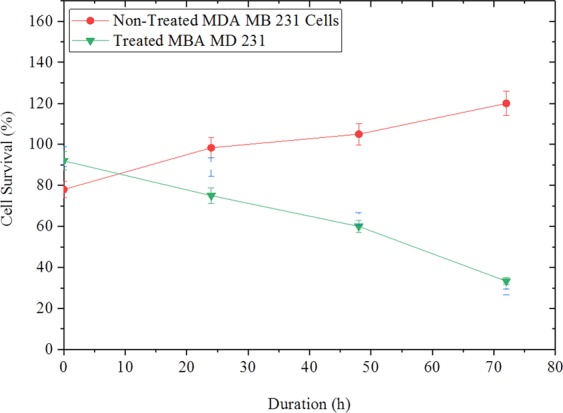
The Survivor Fraction of Cancer and Non-Cancer Cells after Treatment with 8 *nmol*/*ml* PG (the Half Maximum Inhibitory Concentration, IC_50_).

**Figure 8:**
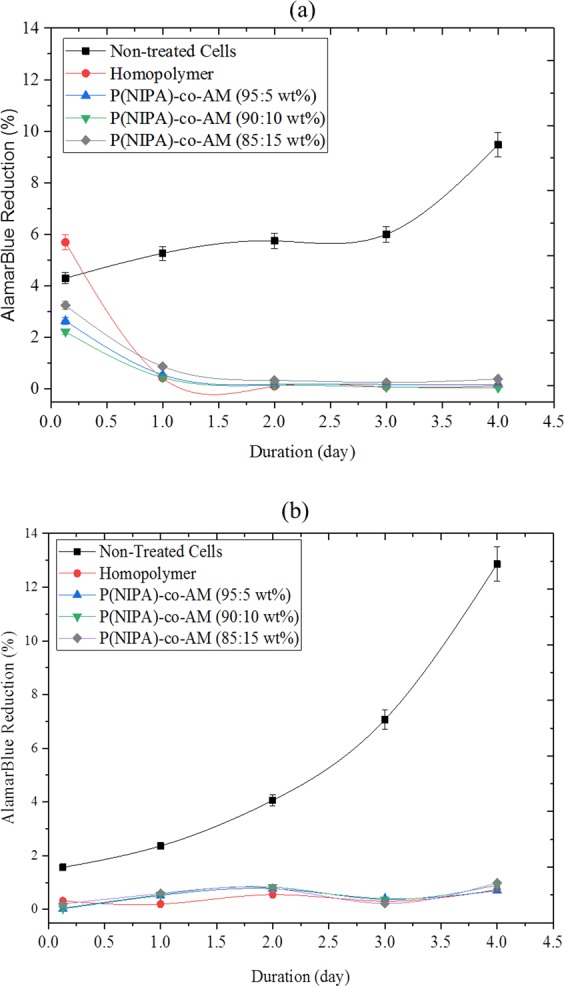
Effect of 8 *nmol*/*ml*PG on the Metabolic Acitivity of: (**a**) Normal Human Cell Line MCF 10A and (**b**) a Breast Cancer Cell Line MDA MB 231, as Shown by the Percentage AlamarBlue Reduction Values.

*In vitro* effect of drug release on cells was described using cells viability study where the drug loaded hydrogels were disinfected and placed in cells seeded well plates. Trypan blue stain was use with the aid of a hemocytometer to estimate the percentage cell survival (survival %) after 72 h period (Fig. 7). The results for MDA MB 231 breast cancer cell line showed an earlier response to drug. This shows that cell survival (%) was more affected in the cancer cell line at the early hours of drug release. The effect of PG release on the metabolic acitivity of normal human cell line (MCF 10A) and breast cancer cell line (MDA MB 231) are presented by the percentage alamarblue reduction as shown in Fig. 8.

## Discussion

The SEM images of the P(NIPA)-based hydrogels showed irregular and heterogeneous mesoporous characteristics structures of the hydrogels (Fig. 1a–d). Prior work has shown that the increased porosity (with increasing AM content) is also associated with increased cross-linking^5–7^. In any case, the porosity of the P(NIPA)-based gels provides pathways for drug diffusion through the P(NIPA)-based structures. This suggests that the presence of these pores affect the resulting swelling ratios. The gel swellings observed prior to de-swelling represents the affinity and exchange of enthalpy between the liquid phase and the solid phase of the gel matrix. A continuous decrease with similar de-swelling rates was observed in Fig. 3a. In contrast, the initial fast de-swelling rates decreased with increasing time at 43 °C (Fig. 3b). However, initially, fast de-swelling rates at 45 °C were followed by a plateau and subsequent fast de-swelling (Fig. 3c). In any case, the amount of de-swelling increased with increasing porosity (Fig. 3a–d) and temperature (Fig. 3a–c).

The slopes and shapes of the bi-dose response curves (S-shape) can provide some insights into the potential effects of specific dose levels of toxicity. The gradients (h_1_ > h_2_) (Tables 4 and 5) from the curves (Fig. 6a) correspond to changes in the amount of drug release. A transient increase in the gradient means an increase in drug concentration and a higher risk of a toxic response and vice versa. It is important to note here that the plotted curves without any initial steepness (A_1_ < A_2_) showed a minimal increase in drug concentration until the concentrations increased progressively with time (Fig. 6b).

Figure 6a indicates a higher threshold dose for P(NIPA)-co-AM (95:5 wt.%), compared to that of P(NIPA)-co-AM (90:10) at 37 °C. Since the higher threshold dose, implies initial effective release, the flow through the P (NIPA) -co-AM (95:5 wt. %) began with a higher dose. However, the slopes increased with increasing concentration. This implies a greater dose response. Hence, P(NIPA)-co-AM (90:10) has a higher dose release than P(NIPA)-co-AM (95:5 wt.%). Similarly, P(NIPA)-co-AM (90:10 wt.%) hydrogel at 43 °C reported a higher threshold dose than P(NIPA)-co-AM (95:5 wt.%) (at 43 °C) (Fig. 6b). In general, an increase in drug release was observed with increasing temperature.

Cell counting with the hemocytometer confirmed cell survival (%) of 33% after 72 h for MDA MB 231. Overall, the results indicated an exponential decrease in cell survival (%) as a function of incubation time. On the other hand, the survival (%) of the treated cells was significantly lower than the control (non-treated) cells (p < 0.05). However, the effect of drug concentration on cell survival is outside the scope of the current paper.

The metabolic activities of non-treated cells increased throughout the 72 h duration of the study for both types of cells. Conversely, the treated cells seeded on the drug-loaded gels exhibited much reduced metabolic activities compared to the non-treated cells, as indicated by the % alamar blue reduction values (Fig. 8a,b). Within the drug-loaded gel groups, there was no clear significant difference due to the polymer or copolymer ratio. However, the results clearly indicated an exponential decrease in % alamar blue reduction value as a function of incubation time (Fig. 8a) and fairly constant values in Fig. 8b. This indirect measurement of cell viability, and by extension, cell growth kinetics kinetics demonstrated that, drug release into the culture medium hindered cell metabolism and growth over time.

From the statistical test that were carried out, there was a mean differences in the metabolic activities, of the non-treated and treated cells with p-values less than 0.05. At the 0.05 level, the difference of the population means was significantly different from the test difference (0). For the MCF 10 A cells, p-values from multiple comparison therefore, suggest statistical significance between the metabolic activities of non-treated cells compared to cells treated with the PG drug. Metabolic activities showed marginal statistical difference between samples treated with drug released from P(NIPA)-co-AM (95:5 wt%) and P(NIPA)-co-AM (90:10 wt%). However, there were no statistical differences when the results of the homopolymer was compared with P(NIPA)-co-AM (95:5 wt%) and P(NIPA)-co-AM (90:10 wt%) p-values were greater than 0.05.

The results obtained from the comparison of untreated MDA MB 231 cells with treated group suggest a statistical significance between the metabolic activities. However, there was no statistical differences based on samples treated with drug released from the different polymer ratios. Moreover, there were no significant differences between the metabolic activities of the normal and cancer cells after treatment over 72 h period.

The implications of the above results are very significant for the controlled delivery of prodigiosin cancer drug^6,7^ to tumor sites^20^. First, they show clearly that there is a need to use the knowledge of the release kinetics to guide the controlled delivery of prodigiosin to be within the therapeutic windows. There is also a need to consider the possible anomalies and super-case transport mechanisms, and the phenomena that could lead to local cytotoxicity during drug release from implantable P(NIPA)-based devices for localized cancer treatment.

Further work is clearly needed to develop implantable drug delivery systems that can deliver cancer drugs, such as prodigiosin, to regions adjacent to tumor sites, following surgery to remove solid tumors from regions such as the breast. Such scenarios could benefit from an implantable device that contains P(NIPA)-based hydrogels that are encapsulated in poly-di-methyl-siloxane (PDMS) packages with micro-channels that deliver the drugs from the P(NIPA) hydrogels to the surrounding tissue^3,6,7^. There is also the potential to explore the combined effects of enhanced cancer drug release and hyperthermia during drug release at 43 and 45 °C^10^. These are clearly some of the opportunities and challenges for future work.

## Conclusions

I. This paper presents the kinetics of drug release from P(NIPA)-based hydrogels that are copolymerized with acrylamide. The swelling and drug release characteristics are elucidated for temperatures in the regime between normal physiological conditions (∼37 °C) and hyperthermic regimes (43–45 °C). Fickian diffusion-controlled release was observed for the P(NIPA)-based homopolymer and P(NIPA)-co-AM (90:10 wt.%) at 37 °C. These have n valuesof 0.45, which correspond to a Fickian diffusion mechanism.
II. The respective release exponents, n, obtained for P(NIPA)-co-AM (95:5 wt.%) at 43 °C was 0.68 and that for P(NIPA)-co-AM (90:10 wt.%) at 43 and 45 °C were 0.56 and 0.67. These n-values correspond to anomalous diffusion mechanisms, with sub-diffusion characteristics. Super case-II transport mechanisms (super-diffusion) were observed during the release of prodigiosin from the P(NIPA)-homopolymer (at 43 °C and 45 °C), P(NIPA)-co-AM (85:15 wt.%) at 43 °C, P(NIPA)-co-AM (95:5 wt.%) at 45 °C, and P(NIPA)-co-AM (85:15 wt.%) at 45 °C. These resulted in n values of 1.30, 1.40, 0.90, 1.06 and 1.03, respectively.
III. In the case of gels that satisfied case-II transport mechanisms, the release characteristics associated with anomalous tended towards those observed typically in the super-diffusion regime in which the release characteristics are controlled by a mixture of swelling-controlled mechanisms and diffusion. Hence, the release rates are time-independent. The diffusion coefficients from the release of the hydrogels range from 6.772 × 10^−8^ to 4.138 × 10^−6^m/s^2^. Diffusion rates were influenced with an increase in application temperature. Drug diffusion was governed by the volume phase transition temperatures. Gels turn to collapse more readily at temperatures above their respective response temperatures. Extensive discussion on the effect of diffusion and the lower critical solution temperature (LCST) on P(NIPA)-based hydrogels, loaded with PG has recently been studied^6^.
IV. The slopes of the bi-dose response curves can be helpful in relating local drug concentrations to therapeutic and toxic levels associated with the release of the prodigiosin cancer drug. In general, a transient increase in the gradient of the bi-dose plots means an increase in the local drug concentration. This corresponds to a higher risk of a local toxic response. However, the plotted curves (without any initial steepness) showed only a minimal increase in drug concentration until the concentrations increased significantly with time.
V. The *in vitro* effect of drug release on cell survival (%) and cell metabolic activities in comparison to the reference (non-treated cells) were presented. The drug inhibited the cell growth of a normal human cell line and a cancer cell line.

## Materials and Experimental Methods

### Materials

The prodigiosin (PG) that was used in this study was obtained from the Biotechnology Advanced Research Center, Sheda Science and Technology Complex (SHESTCO), Abuja, Nigeria. The P(NIPA)-based gels were synthesized from N-Isopropylacrylamide (NIPA), 97% (the main monomer), N,N,N′,N′-Tetramethyl-ethylene-diamine (TEMED), 99% (used to accelerate the rate of addition polymerization), N,N′-Methylene-bis-acrylamide (MBA), 99% (a cross-linking agent), ammonium persulfate (APS), 98% (a radical initiator), and acrylamide (AM), 99.9% (a co-monomer). These constituents were all purchased from the Sigma Aldrich Company (St. Louis, MO, USA). Human breast cell line (MCF 10A) and human breast carcinoma cell line MDA-MB-231 were obtained from the American Type Culture Collection (Manassas, VA, USA). Liebovitz’s 15 medium, fetal bovine serum (FBS), penicillin/streptomycin antibiotics, 0.25% trypsin-EDTA and DMEM/Ham’s F12 medium (1:1) were procured from Gibco Thermo Fisher Scientific (Waltham, MA, USA).

### Polymerization of P(NIPA)-Based Hydrogels

**Figure 9:**
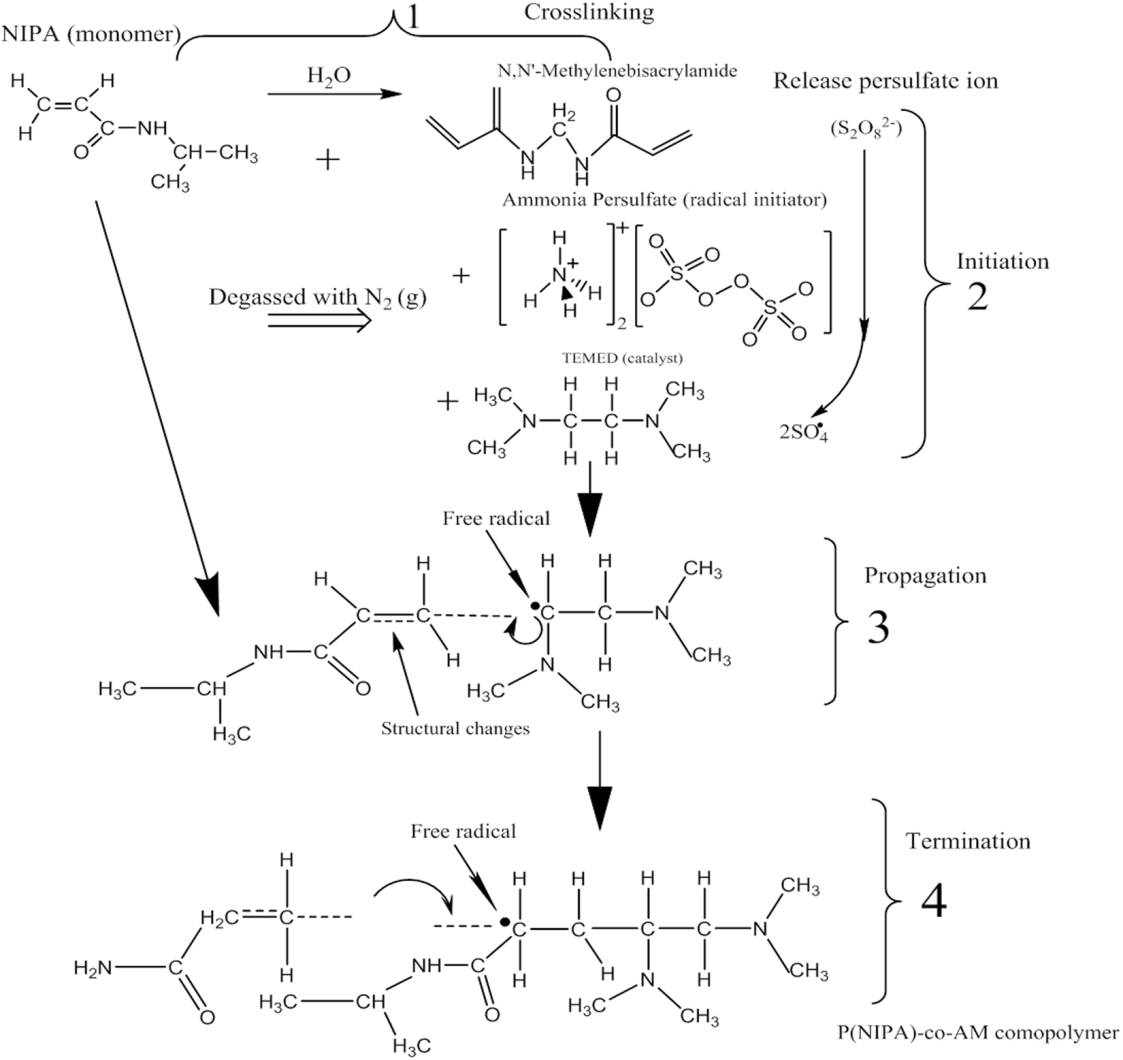
Crosslinking and Free Radical Polymerization of P(NIPA)-Based Hydrogels.

Polymer samples were weighed to desired quantities. This comprised of 3.48 g NIPA, 0.134 g APS, 0.0544 g MBA which were subsequently dissolved vigorosily in 31.2 ml double deionized water. The resulting samples were immersed and cooled down in ice to control polymerization temperatures. MBA is used as a cross-linker because of its two acrylamide molecules linked by a methylene group. APS is used as a radical initiator, while 40 *μl* of TEMED was used as a catalyst via gently swirling the mixture to decompose persulfate ions release, to give free radicals (molecules with an unpaired electrons) (Fig. 9). The unpaired electron then continues to develop into macromolecules, especially when MBA is crosslinked with AM. After the addition and reaction of TEMEDwith the gel solution, the polymerisation process of P(NIPA)-based hydrogels were then terminated within 5–10 s via the interaction of the gel solutions with oxygen radicals in the environment. That is, the gel solutions in test tubes were completely openedand samples were exposed to oxygen to terminate the reactions and solid gels were obtained within 3 mins.

Hence, polymerization of P(NIPA)-based hydrogels was carried out by free radicals polymerization as presented in our prior work^7^. The P(NIPA)-based co-polymers were polymerized with the AM monomer to produce P(NIPA)-based co-polymers with 5, 10 and 15 wt.% of acrylamide. These correspond to: P(NIPA)-co-AM (95:5 wt.%); P(NIPA)-co-AM (90:10 wt.%), and P(NIPA)-co-AM (85:15 wt.%), respectively. Summary of the methodology on the gel polymerization is presented (Fig. 9).

The polymerized hydrogels were subject to several washes, soaking and de-swelling in double deionized water at room temperature to remove, through leaching, any unreacted acrylamide or chemicals salts. A sudden corrosion of the stainless steel razor blades that were used to slice the gels was indicative of the presence of unreacted chemicals/chemical residue in the polymerized gels and this was not observed after thoroughly washing the gels.

The gels were then sliced into the required thicknesses/geometries, washed and dried under vacuum with an isotemp vacuum oven (model 280A, Fisher Scientific, Waltham, MA, USA), connected to a high vacuum pump (Edwards, E2M265503) set at −20 in Hg for 2 h at 40 °C to drive out excess water. The water release from the samples was constantly discarded. Subsequently, gels were completely dried at room temperature until constant weights were achieved, prior to soaking them in drug solutions. Though drying had the tendency to collapse the gels, P(NIPA)-based hydrogels have the ability to recover their natural porosity by expanding, stretching and uncoiling their folded chains when soaked in fluids/drug solutions.

### De-Swelling Kinetics

The drug affinity, hydrophilicity, and swelling of the hydrogels were studied by incubating dried gels (cylindrical structures) in drug solutions (PG). This was carried out in an orbital incubator shaker (Innova 4300, New Brunswick Company Inc. NJ, USA) at a controlled temperature (27 °C). Incubation was controlled until an equilibrium swollen mass ratio was obtained. This occurred within 72 h of incubation. De-swelling experimentswere then carried out in an orbital incubator shaker, operated at physiological conditions (37 °C at 60 rpm). De-swelling experiments were also performed at 43 and 45 °C to study possible effects of hyperthermic temperatures on drug release kinetics. The de-swelling ratios (DSR) of prodigiosin from P(NIPA)-based hydrogels were obtained from the expression:

2 $$DSR=({M}_{eq}-{M}_{t})/{M}_{eq}$$

where M_t_ is the mass of a hydrogel at a time, t, and M_eq_ is the equilibrium mass of the swollen hydrogel. The percentage water absorbability (swelling ratio, %) is given by^21^:

3 $$WA=[({M}_{a}-{M}_{d})/{M}_{a}]\times 100$$

where M_d_ is the dry mass of a hydrogel at time t = 0 and M_a_ is the mass of a hydrogel in water after 72 h. The hydrophobicity was determined at the equilibrium swelling in deionized water (*S*_*eq*_). This is given by:

4 $${S}_{eq({H}_{2}0)}={M}_{s}/{M}_{d}$$

where *M*_*s*_ is the swollen mass and *M*_*d*_ is the dry mass of the gel after soaking in 1 ml of distilled water for 72 h. The hydrophilic swelling index (H-index) for the gels was obtained from:

5 $$H-index=[\frac{{S}_{eq}(70\,vol \% \,Is)}{{S}_{eq}({H}_{2}O)}]\times 100$$

where S_eq_(70 vol.% Is) is the equilibrium swelling mass of a gel in aqueous 70% iso-propanol for 72 h.

### Drug Release Under Physiological Conditions

P(NIPA)-based hydrogels were loaded with prepared concentrations of prodigiosin (PG). Knowledge from the molecular weight of drug candidate was used to prepare the desired drug concentrations (8 nM/ml) for loading the hydrogels via incubation at room temperature for 72 h to ensure complete saturation. The prepared concentration of drugs was also guided by existing literature^21,22^. Drug release from P(NIPA)-based hydrogels were conducted under *in vitro* conditions at 37 °C under a mechanical agitation of 60 rpm in a phosphate buffer saline (PBS) solution, pH 7.4. These conditions were considered to simulate physiological conditions. Ultraviolet-visible (UV-Vis) spectrophotometer (Evolution 300, Shimadzu Scientific Instruments Inc., Kyoto, Japan) was used to characterize the absorbance of drug release. Concentrations were obtained from the absorbance’s values reported. Drug release from P(NIPA)-based hydrogels usually starts with a rapid release. Due to this, 1 ml of drug solutions were collected at 10 min interval on the first day of drug elution, while daily decanting and replacement with equal volume was done.

### Mechanisms of Drug Release

The fraction of drug release, *M*_*t*_/*M*_*o*_, exhibits a power law dependence on time, t, which is given by^23,24^:

6a $$\frac{{m}_{t}}{{m}_{o}}=k{t}^{n}$$

where $\,\frac{{m}_{t}}{{m}_{o}}$ is the fraction of drug release at time t, k is a geometry constant, and n is the release exponent, which corresponds to the mechanism of drug diffusion.

The constants k and n were obtained from the linear logarithm form of equation (6a). This gives:

6b $$ln\,({m}_{t}/{m}_{o})=\,\mathrm{log}\,k+n\,\mathrm{log}\,t$$

where the values of k and n were obtained from the intercepts and gradients of the plots of ln(*m*_*t*_/m_o_) versus ln t(s). Meanwhile, the diffusion coefficients, D, were obtained from:

7 $$D=\frac{k\pi {\delta }^{2}}{4}$$

where *δ* is the thickness of the dry gel and k is the constant in Equation 6a.

A recent study^6^, including the current work, suggest a dominance of anomalous transport mechanisms with 0.45 < n < 0.89, for cylindrical disc specimens^23,25,26^. These correspond to a regime in which non-Fickian diffusion control the drug release. Since such anomalous drug release is less well understood than conventional release, there is a need for further work to improve our basic understanding from P(NIPA)-based gel. In the current study, sigmoidal and two-step type of anomalous behaviours were investigated by plotting $\frac{{m}_{t}}{{m}_{o}}$ versus $\sqrt{t}$, while case-II transport mechanisms were also investigated by plotting $\frac{{m}_{t}}{{m}_{o}}$ versus t. Finally, the classical transport mechanism was explored using plot of $\frac{{m}_{t}}{{m}_{o}}$ versus $\frac{\sqrt{t}}{L}$.

The above plots were obtained using OriginPro2017 software package (OriginLab Corporation, Northampton, MA, USA)^27^. The regression coefficient, R^2^, was used as the goodness of fit in the studies of the anomalous behavior. Four kinetic models were used to characterize the release kinetics. This includes zeroth-order, first order, second order, Higuchi model and Korsmeyer-Peppas equation (Table 6). These were used to fit the data obtained from the drug release experiments^28^.

**Table 6:** Summary of Equations for Different Drug Release Kinetics.

Model	Equation	Constant
Zeroth-order	*C*_*t*_ = *C*_*o*_ − *k*_*o*_*t*	*C*_*t*_ is the amount of drug release (assuming the release occurs rapidly) after a time, t; *C*_*o*_ is the initial amount of drug; *k*_*o*_ is the Zero-order rate constant.
First-order	*ln C*_*t*_ = *ln C*_*o*_ − *k*_*I*_*t*	*C*_*t*_ is the amount of drug release after time, t; *C*_*o*_ is the initial concentration of drug; *k*_*I*_ is the First-order constant.
Second-order	$\frac{1}{{C}_{t}}=\frac{1}{{C}_{o}}+{k}_{II}t$	*C*_*t*_ is the aa mount of drug release after time, t; *C*_*o*_ is the initial concentration of drug; *k*_*II*_ is the Second-order constant.
Higuichi	*C*_*t*_ = [*D*(2*C* − *C*_*s*_*t*)]^1/2^	*C*_*t*_ is the amount of drug release after time, t; *C* is the initial drug concentration; *C*_*s*_ is the drug solubility in the matrix media; D is the diffusivity of drug molecules in the matrix substance.
Korsmeyer-Peppas equation	$\frac{{m}_{t}}{{m}_{o}}=k{t}^{n}\,$	$\frac{{m}_{t}}{{m}_{o}}$ is the fraction of drug release at time t, k is a geometry constant, and n is the release exponent which corresponds to the mechanism of drug diffusion.

### Material Characterization

Prior to acquiring morphological characteristics with scanning electron microscopy (SEM), sample preparation involved sputter-coated P(NIPA)-based hydrogels with 4 nm of gold under vacuum using a sputter coater (EMS Quorum, EMS150R ES, Quorum Technologies Ltd., East Sussex, UK). Samples were then mounted on standard SEM sample holder, gently loaded into Phenom ProX desktop SEM (ThermoFisher Scientific, Phenom-World B.V., Eindhoven, The Netharlands). Images were acquired and morphological differences were observed and reported. Pore diameters were determined with ImageJ software package (2015 version)^29^.

Thermal analysis on the lower critical solution temperature (phase transition temperature) withdifferential scanning calorimetric (DSC) analysis was carried out by loading ~10 *mg* of each sample into an aluminum pan. The mass of the reference sample (empty Al pan) was equally measured. Samples were loaded onto DSC (214 Polyma, Netzsch Instruments, Burlington, MA, USA). Methods for data acquisition and analysis were defined with the instrument control software at a heating rate of 10 °C/min. Data acquisition was done in a nitrogen environment by purging N_2_ at a flow rate of 20 ml/min. DSC was done from room temperature (26 °C) to 60 °C to determine the LCST.

### ***In Vitro*** Effect of Drugs Release on Cell Survival Fraction

Prior to cell culture, P(NIPA)-based hydrogels were disinfected with ethanol. Samples were dipped completely into 70% ethanol untill saturation for 1 h. To ensure a complete removal of the ethanol (which can affect cell viability), the hydrogels were first cleaned ultrasonically for 30 min with autoclaved double deionized water. The samples were also rinsed several times with Dulbecco’s phosphate buffered saline (DPBS). The disinfected samples were allowed to dry at room temperature while being exposed to UV light under sterile conditions in a biosafety cabinet before they were subsequently loaded with 8 nmol/ml drug solutions (PG). Samples were also exposed to UV light prior to cell seeding.

Human breast cancer cell line (MDA-MB-231) was used to investigate the effects of the free PG drug and drug released from P(NIPA)-based hydrogels on cell survival (%). The MDA-MB-231 cell line were cultured with Liebovitz’s L15 medium supplemented with 10% fetal bovine serum (FBS) and 1% penicillin/streptomycin antibiotic. MCF 10A cell lines were cultured with DMEM/F12 medium that were supplemented with 0.5 *μg*/*ml* hyrocortisone, 5% horse serum, 10 *μg*/*ml* insulin, 30 ng/ml murine Epidermal Growth Factor, 100 ng/ml cholera toxin, 0.2% amphotericin and 1% Penicillin-Streptomycin. The MCF 10A cell lines were cultured in T75 flask at 37 °C in a humidified incubator 5% CO_2_, whereas the MDA-MB-231 cell lines were in T75 tissue culture flask at 37 °C until 80–90% confluence (log phase of growth) was achieved. The cells were then harvested with 0.25% rypsin-EDTA and 10,000 cells/well were seeded in 24-well plates (n = 4). After 48 h, the cells attained their 70% confluence state (log phase growth). Then, a concentration of 8 nmol/ml of PG drug prepared in the culture medium was added to the wells with 10,000 cells/well (treated cell wells). Cells in wells without drugs were used as control wells with 10,000 cells/well (non-treated cell-wells). The cells were immediately trypsinized and stained at different time just after 6 h, 24 h, 48 h, 72 h, and 96 h with trypan blue before counted with a hemocytometer. The percentage cell survival (%)/cell viability (%) was calculated from:

8 $$ \% \,Survival/Cell\,Viability=\frac{Number\,of\,Live\,Cells}{Total\,number\,of\,cells\,}\times 100$$

where the total number of cells equals to the number of live plus dead cells counted.

### *In Vitro* Effect of Drugs Release on Cell Metabolic Activities

AlamarBlue assay, a fluorometric method for the detection of metabolic activity of cells, was used as an indirect measure of cell viability. MCF 10A and MDA MB 231 cells in their log phase of growth were harvested with trypsin-EDTA and 10,000 cells/ml were seeded on the various PG drug-loaded hydrogels in 24-wells plates (treated groups) and on non-loaded gels (non-treated control) (n = 3 per group). After a 3 h cell attachedment period (time 0), the culture medium was replaced with 1 ml of culture medium containing 10% alamarBlue reagent (Thermo Fisher Scientifi). The plates were incubated in a humidified incubator at 37 °C for 3 h and 100 *μl* aliquots were transferred into duplicate wells of a black opaque 96-well plate (Thermo Fisher Scientific). The fluorescence intensities were then measured at 544 nm excitation and 590 nm emission using a 1420 Victor3 multilable plate reader (Perkin Elmer, Waltham, MA). All alamarBlue reagents were prepared according to the manufacturer’s instructions. The percentage reduction of alamarBlue reagent was determined using equation (9).

9 $$ \% \,Reduction\,of\,AlamarBlue=\frac{F{I}_{sample}-F{I}_{10 \% AB}}{F{I}_{100 \% R}-F{I}_{10 \% AB}}\times 100$$

where FI_sample_ is the experimental fluorescence intensity of samples, FI_10%AB_ is the fluorescent intensity of the 10% alamarBlue (negative control: oxidized form of alamarBlue), and FI_100%R_ is the fluorescence intensity of 100% reduced form of alamarBlue (positive control).

### Statistical Analysis

Statistical analysis was carried out with OriginPro 2017 software package. One-way ANOVA and paired sample *t*-test were used for multiple comparisons. Statistical significance in the survival (%) of treated and non-treated cancer cells were statistically evaluated based on the differences in population means. The difference in toxicity obtained for free drug and the drug released from the hydrogels was also evaluated. Thus, p < 0.05 was considered to bestatistically significant, unless otherwise stated.

## Supplementary information

Anomalous Release Kinetics of Prodigiosin from Poly-N-Isopropyl-Acrylamid based Hydrogels for The Treatment of Triple Negative Breast Cancer
