# Supplementary material for: Anomalous Release Kinetics of Prodigiosin from Poly-N-Isopropyl-Acrylamid based Hydrogels for The Treatment of Triple Negative Breast Cancer
Source: Sci Rep. 2019 Mar 7;9:3862. doi: 10.1038/s41598-019-39578-4 (PMC6405774; doi:10.1038/s41598-019-39578-4)
Supplement: Supplementary file 1 — Anomalous Release Kinetics of Prodigiosin from Poly-N-Isopropyl-Acrylamid based Hydrogels for The Treatment of Triple Negative Breast Cancer [file 41598_2019_39578_MOESM1_ESM.pdf]

Appendix A

Anomalous release kinetics of prodigiosin from poly-n-isopropyl-acrylamide-based hydrogels for the treatment of triple negative breast cancer

Y. Danyuo, C. J.Ani , A. A. Salifu, J. D. Obayemi, S. Dozie-Nwachukwu, V. O. Obanawu, U. M. Akpan, O. S. Odusanya, M. Abade-Abugre, F. McBagonluri and W. O. Soboyejo.

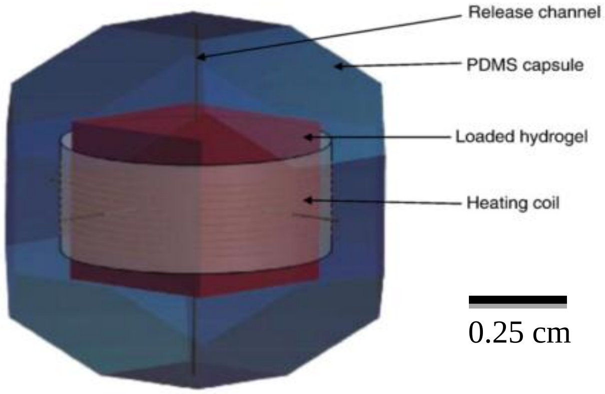

Schematics of the multi-modal device. Reused with permission (with License Number4422250215514 and License date Sep 04, 2018)from Oni et al. [3], Materials Science and Engineering C, published by Elsevier.

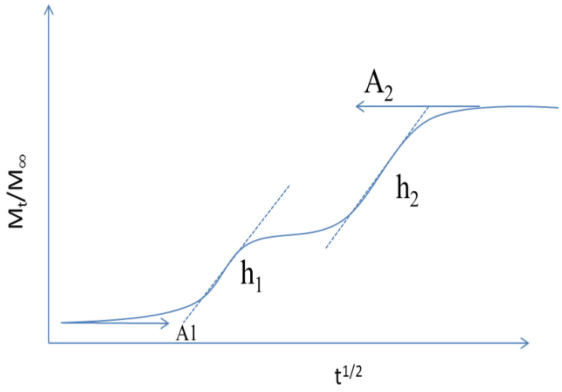

Illustration of Parameters for Bi-Dose Response for a Sigmodal Class of Anomalous Transport.
